# Behavioral, physiological, encephalopathological alterations and endoplasmic reticulum stress/apoptotic-related gene transcription in reaction to heat stress in *Oreochromis niloticus* fish: impact of dietary supplements with Spirulina-Coenzyme Q10 nanoemulsion

**DOI:** 10.1007/s11259-025-11017-y

**Published:** 2026-01-21

**Authors:** Walaa El-Houseiny, Mohamed Elhady, Shaimaa A. A. Ahmed, Tarek Khamis, Sameh H. Ismail, Mohamed M.M. Metwally, Wessam El-Shahat

**Affiliations:** 1 Department of Aquatic Animal Medicine, Faculty of Veterinary Medicine, Zagazig University, Zagazig, 44511 Egypt; 2 Department of Pharmacology, Faculty of Veterinary Medicine, Zagazig University, Zagazig, 44511 Egypt; 3 Laboratory of Biotechnology, Faculty of Veterinary Medicine, Zagazig University, Zagazig, 44511 Egypt; 4 Faculty of Nanotechnology for Postgraduate Studies, Sheikh Zayed Branch Campus, Cairo University, Sheikh Zayed City, Giza, 12588 Egypt; 5 Department of Pathology, Faculty of Veterinary Medicine, Zagazig University, Sharkia, Zagazig, 44511 Egypt

**Keywords:** Spirulina-co-enzyme Q10 nanoemulsion, Behavior, Heat stress, Heat shock protein, Apoptosis

## Abstract

Water temperature significantly influences fish behavior and physiology. Thus, the current study assessed the effects of dietary incorporation of Spirulina-co-enzyme Q10 nanoemulsion (SCN) on various health parameters in Nile tilapia (*Oreochromis niloticus*) raised in heat stress conditions (32 °C) for 60 days. In a completely randomized design, Nile tilapia (*n* = 225) were divided into 5 groups (3 replicates, 15 fish per group). The first (C25) and second (SCN0) groups were fed a basal diet and reared at 25 °C and 32 °C, respectively. Groups 3–5 were reared at 32 °C and fed a diet supplemented with SCN at 10 mg/kg (SCN10), 20 mg/kg (SCN20), and 40 mg/kg (SCN40), respectively. The findings indicated that the inclusion of SCN in the diets of *O. niloticus*, particularly at 20 and 40 mg/ kg concentrations, significantly (*P <* 0.05) counteracted the heat stress (32 °C) impact on behavior, hematological parameters, stress biochemical indicators, additionally stress related genes and histopathological changes in fish, were assessed. SCN0 expressed decline in feeding frequency and mid-water swimming behavior, surfacing and high opercular movement. Cholesterol, triglycerides, glucose, and cortisol levels were significantly decresead with SCN supplementation. Heat stress induced significant reduction (*p <* 0.05) in the hematological indices (RBCs, WBCs, Hb, and HCT%), which were ameriolated after SCN addition. SCN also significantly decreased encephalopathic alterations (vascular congestion, perivascular edema, neuronal pyknosis associated with perineuronal vacuolation, and neuropil microcavitation) in the brain of fish treated at 32 °C. The incorporation of SCN into the diet markedly prevented the abnormal expression of *hsp70*,* tgf****-****β*,* p53*,* Chop*, and *Bip* in the brain of heat-stressed fish. In conclusion, SCN at rate of 20 mg/ kg is a promising dietary supplement capable of mitigating the negative effects of heat stress in Nile tilapia.

**Supplementary Information:**

The online version contains supplementary material available at 10.1007/s11259-025-11017-y.

## Introduction

Aquaculture is a rapidly expanding food-producing industry that supplies millions of people worldwide with fish protein. Given the predicted population growth and the significant percentage of undernourished individuals, the production of fish needs to be expanded to meet the demand (Bjørndal et al. 2024). One of the primary aquaculture species is the Nile tilapia (*Oreochromis niloticus*), which ranks as the third world’s most cultivated fish (4407.2 thousand tons, or 9% of the total volume) (FAO 2022). This species has remarkable adaptations, including resistance to a broad range of environmental stresses, fast development, effective reproductive mechanisms, and feeding at different trophic levels (Peterson 2005).

Elevated temperatures and severe weather linked to global warming impose a growing stress on fish. Moreover, under intensive production systems, fish are persistently exposed to multiple stressors. Specifically, heat stress can disrupt the body’s physiological balance, which might hinder development and survival (Ibrahim et al. 2019). The ideal temperature range for the growth and survival of *O. niloticus* is between 25 and 28 °C (Mahmoud et al. 2020). High water temperatures (32 °C) induced notable stunting of growth and immunosuppression, decreased the activity of antioxidant enzymes, and altered cortisol levels (Ibrahim et al. 2019). Nile tilapia reared at 32 °C suffered from multiple stress symptoms, including reactions to decreased dissolved oxygen levels, reduced appetite, and surfacing (Said et al. 2020; Jinagool et al. 2024; Mekonnen et al. 2025). Moreover, the expression of *hsp70* and endoplasmic reticulum stress-associated genes (*chop*) was high in response to heat stress (Hassan et al. 2017). Considering the multiple responses of Nile tilapia to heat stress, comprehensive protocols, incorporating all these responses, are needed to assess the effects of environmental challenges.

The brain tissue in fish is particularly vulnerable to oxidative stress (heat stress) since it is a thermosensitive organ with limited antioxidant defense. The fish brain is responsible for recognizing, controlling, and adjusting to temperature variations (Nonnis et al. 2021). This extended exposure to high temperatures may lead to metabolic and inflammatory dysfunctions in the tissues (Alfons et al. 2023). It sustains physiological equilibrium by a variety of processes, including sensing temperature, neuroendocrine regulation, neurological protection, behavior manipulation via neurotransmitters, and persistent adaptation via the control of gene expression (Haesemeyer et al. 2018). Several investigations manipulate the relation between heat stress and the brain in different fish species (Guan et al. 2025; Zhao et al. 2024; Zhang et al. 2022; Topal et al. 2021). Transcriptomic studies in fish species (e.g., Chinese tongue sole, *Trematomus bernacchii*, Atlantic salmon) showed that elevated temperature or restricted thermal ranges provoke significant alterations in brain gene expression including neural- and behavior-relevant pathways (Wang et al. 2023; Beemelmanns et al. 2021).

Recently, there has been a growing interest in the integration of natural supplements into tilapia diets to mitigate hyperthermia stress. Nanotechnology is a promising field in aquaculture in order to create beneficial nano-sized compounds that can be used as feed additives and therapeutic agents (El-Houseiny et al. 2021). *Spirulina platensis* is a type of blue-green algae that comprises various dietary constituents, including protein, B vitamins (predominantly riboflavin), essential minerals (notably iron), essential amino acids, polyunsaturated fatty acids (PUFA), chlorophyll, carotenoids, and specific pigments (such as phycocyanin and allophycocyanin) (Güroy et al. 2012). These constituents can neutralize free radicals and fight against oxidative stress. Nanoparticles are renowned for their distinctive properties and capacity to linger in the bloodstream for extended durations, enhancing their accessibility and efficacy (Hill and Li 2017). Coenzyme Q10 is an effective free radical scavenging agent, naturally found in most living cells’ mitochondria (Eleiwa et al. 2024). It not only promotes vitamin regeneration but also protects the cell membranes, lipids, proteins, and DNA against oxidative damage (Yubero-Serrano et al. 2020). In addition, it has a powerful role as an antioxidant agent, and hence, efficiently aids in mitigating oxidative stress (El Basuini et al. 2021; Khalil et al. 2022). It also preserves the integrity and function of neurons by acting as a neuroprotectant, which stops the cell death cascade in its tracks (Li et al. 2016). Finally, Coenzyme Q10 nanoparticles help circumvent the issues of poor absorption and restricted bioavailability of CoQ10 (Amit et al. 2022). The bioactivity compounds found in *Spirulina platensis* discussed above, and Coenzyme Q10 likely have synergistic effects when used together. Their combination in diets may therefore offer even better improvements of Nile tilapia to heat stress but has not been investigated to date.

Accordingly, this work used behavioral observations, physiological traits including expression of heat stress-related genes, and brain histology to evaluate the potentially harmful effects of heat stress exposure on Nile tilapia (*Oreochromis niloticus*) and the ameliorative effects of SCN on these responses.

## Materials and methods

### Preparation and characterization of spirulina-Coenzyme Q10 nanoemulsion (SCN)

High-quality, pharmaceutical-grade materials were utilized for the preparation. Spirulina powder (*Arthrospira platensis*) was sourced from the National Research Center, Giza, Egypt, and verified for purity and potency through spectrophotometric analysis. Coenzyme Q10 (Co-Q10) was bought from Mepaco-Medifood Co. (Sharkia, Egypt). The synthesis of the Spirulina-Coenzyme Q10 nanoemulsion was conducted using an aqueous-based nanoemulsion technique. The entire synthesis process was carried out in a laminar flow hood to maintain aseptic conditions throughout the preparation. The formulation consisted of three primary components in a volume ratio of 1:2:3, where 1 part was extra virgin olive oil (oil phase), 2 parts were Tween 80 (non-ionic surfactant), and 3 parts were purified water (aqueous phase). The next step was to make a coarse emulsion by slowly adding the oil phase to the water phase (weight/weight) and mixing it extremely well under continuous high-speed homogenization (20,000 rpm, 30 min) (High-speed homogenizer: IKA T25 digital ULTRA-TURRAX). This coarse emulsion was then subjected to ultrasonication using a probe sonicator (Model: Sonics Vibra-Cell VCX 750, Sonics & Materials Inc., USA), at a frequency = 20 kHz with the highest power output at 750 W. Then exposed to an ice bath to prevent thermal degradation of components. All characterization measurements were performed in triplicate (*n* = 3) to ensure reproducibility. Statistical analysis was performed using one-way ANOVA, and results are presented as mean ± standard deviation.

### Characterization of SCN

Atomic Force Microscopy (AFM) Analysis was employed to obtain three-dimensional topographical images of the nanoemulsions and to measure their mechanical properties at the nanoscale (Bruker Dimension Icon ICON-PT-2 (Bruker Corp., Santa Barbara, CA, USA). The nanomechanical properties revealed by AFM, such as elasticity and adhesion, provide insights into the behavior of the nanoemulsions under physiological conditions, helping to predict their stability and interaction with biological barriers. A Rigaku Smart Lab X-ray Diffractometer instrument (Rigaku SmartLab X-ray Diffractometer (Rigaku SmartLab XRD-6100 (Rigaku Corp., Tokyo, Japan) was used to investigate the crystalline structure of the Spirulina powder and to detect any potential crystalline forms of the encapsulated Co-enzyme within the nanoemulsions. The crystallinity information is crucial for predicting the dissolution behavior and bioavailability of the active compounds in vivo, which directly influences their therapeutic efficacy in biomedical applications. XRD was conducted using a 2θ range of 5° to 60° with a step size of 0.02° and a scan rate of 2°/min. The working current was 30 mA at 40 kV. Transmission Electron Microscopy (TEM) imaging was used to visualize the morphology and internal structure of the nanoemulsion droplets at high resolution using a JEOL JEM-2100 F (JEOL Ltd., Tokyo, Japan) Field Emission Electron Microscope, providing direct evidence of successful encapsulation. Detailed structural information helps in optimizing the formulation process and predicting the behavior of the nanoemulsions in biological environments, which is essential for their efficacy in drug delivery applications. Dynamic Light Scattering (DLS) measurements were conducted to determine the hydrodynamic diameter and size distribution of the nanoemulsion droplets using a Malvern Zetasizer Nano ZS (Malvern Instruments Ltd., Worcestershire, UK). This technique provides insight into the uniformity and stability of the formulation. The Zeta Potential was determined using a Malvern Zetasizer Nano ZS (Malvern Instruments Ltd., Worcestershire, UK). This analysis provided an assessment of the surface charge of the nanoemulsion droplets, which is a key indicator of colloidal stability. A high absolute zeta potential value (typically >|30| mV) suggests enhanced stability against aggregation, which is crucial for maintaining the shelf-life and in vivo performance of the nanoemulsions in biomedical applications. Measurements were performed at 25 °C with a scattering angle of 173° and a dielectric constant of 78.5.

### Fish rearing conditions

Nile tilapia were procured from a private fish farm in Abbassa, Abo-Hammad, Sharkia, Egypt. The experimental trial was subsequently conducted in the laboratory of the Department of Aquatic Animal Medicine, Faculty of Veterinary Medicine. Two weeks before the start of the experiment, fish were acclimated to lab conditions in 100 L capacity glass aquariums that were thermostatically controlled and supplied with tap water dechlorinated using aerators. Throughout the trial, the following water parameters were monitored every day and maintained within the suitable ranges for tilapia aquaculture (APHA 1998. The temperature within each tank was set to 25 °C and 32 °C using a thermostat heater (Sea Star HX-906, 50–60 Hz, 20–32 °C, 150 W). We used an oxygen meter (970 portable DO meter, Jenway, London, UK) to monitor the dissolved oxygen (DO) level. The Digital Mini-pH meter, model 55, manufactured by Fisher Scientific in Denver, Colorado, USA, was used to measure the pH. The HC HI733 (HANNA, Egypt) high-range ammonia colorimeter was used to measure the ammonia level. Using API nitrite test kits (API Fish Care, Egypt), the nitrite concentration was analyzed. The DO levels were maintained at 6.6 ± 0.5 mg/L, water temperature was kept at 25 ± 0.5 °C or 32 ± 0.6 °C, Ammonia NH3 at 0.02 ± 0.01, and pH was maintained at 7.2 ± 0.5.

### Outline of the experiment and diet planning

The base diet used during the experiment was designed to meet Nile tilapia’s essential nutrient requirements, as recorded by the National Research Council (NRC 2011). Procedures to prepare the base diet were previously described by Ayyat et al. (2024), and the composition of the diet is given in Table 1. After acclimatization, 225 Nile tilapia (35.99 ± 0.59 g mean starting weight) were randomly distributed into five experimental groups (3 replicates per group, 15 fish per replicate). The experiment was applied for 8 weeks by rearing the fish under two different temperatures (25 °C and 32 °C). The first group (C25) represented a negative control, reared at 25°Cand fed the base diet. The second group (SCN0) represented a positive control, reared at 32°Cand fed the base diet. The 3rd −5th groups were reared at 32 °C and fed on the base diet supplemented with different concentrations of SCN: 10 mg/kg (SCN10), 20 mg/kg (SCN20), and 40 mg/kg (SCN40), respectively. In both the acclimatization and experimental stages, the fish were given a basal diet of 3% of their body weight daily. The feeding schedule of the Nile tilapia was designed to mimic its natural diurnal feeding patterns, with presentations at 9:00 and 15:00 h each day. According to research by Hamed et al. (2024) and Souza et al. (2014), tilapia show their highest eating activity around mid-morning and mid-afternoon under regular photoperiods. This suggests that these times are ideal for maximizing feed intake efficiency and growth performance in Tilapia fish. To make sure the fish had enough nutrients without being overfed, they were fed until they obviously felt full. By measuring and weighing a representative subset of fish from each group, changes in biomass were used to alter the feeding amount every two weeks. Daily, the uneaten feed and excrement were removed using a siphoning method to keep the water quality optimal and to reduce waste accumulation. Also, every other day, half of the water in each tank was replaced. In order to prevent stress caused by abrupt changes in environmental conditions, this partial water exchange was executed with great care using pre-aerated, temperature-matched, dechlorinated running water. Throughout the experiment, the water temperature of each treatment was monitored continuously using a thermostat heater (Sea Star HX-906, 50–60 Hz, 20–32 °C, 150 W) to ensure that the temperature was maintained at 25 ± 0.5 °C or 32 ± 0.6 °C. Oxygen saturation was maintained using continuous aeration.

**Table 1:** Experimental diet ingredients and proximate chemical analysis

Ingredients	g/kg
Fish meal, 60%	120
Soybean meal 44%	410
Ground corn	230
Corn oil	50
Wheat bran	100
Cod liver oil	20
starch	40
Vitamin premix^1^	10
Mineral premix^2^	20
Total	1000
Chemical analysis	
Crude protein (*N* ×6.25)	30.4
Crude lipids	7.42
Crude fiber	5.25
Ash	7.24
Nitrogen free extract^3^	49.69
Gross energy (kcal/kg) ^4^	446.104

^1^ Vitamin premix (per kg of premix): vitamin A,8000000 IU; vitamin E, 7000 mg; vitamin D_3_, 2000000 IU; vitamin K_3_,1500 mg; biotin, 50 mg; folic acid, 700 mg; nicotinic, 20000 mg; pantothenic acid,7000 mg; vitamin B_1_, 700 mg; vitamin B_2_, 3500 mg; vitamin B_6_, 1000 mg; vitamin B_12_, 7 mg.

^2^ Mineral premix (per kg of premix): zinc sulfate, 4.0 g; iron sulfate, 20 g; manganese sulfate,5.3 g; copper sulfate, 2.7 g; calcium iodine, 0.34 g; sodium selenite, 70 mg; cobalt sulfate, 70 mg, and CaHPO_4_·2H_2_O up to 1 kg.

^3^ Calculated by difference (100 –protein% + lipids%+ ash% + crude fiber %).

^4^ Gross energy (GE) was calculated as 5.65, 9.45 and 4.11 kcal/g for protein, lipid and NFE, respectively.

### Evaluation of behavioral alterations

All behavioral attitudes were observed using scan observation for each group twice daily 15 min/period (5 min/replicate) over 6 h daily using a stopwatch at circulatory predetermined time (Altmann 1974).

Aggressive conduct: According to De Boer (1980), this behavior includes a variety of patterns, including mouth pushing (two fish standing against each other with their mouths open), chasing (fish violently pursue each other), and approach (direct movement toward another fish).

Swimming behavior: This refers to the fish’s swimming style, which can be either fast or slow, lacking any discernible behavioral activity in the bottom, middle, or surface of the tank (Chen et al. 2001).

Resting behavior: According to Scheurmann (2000), this is the behavior in which fish sit motionless at the bottom of the aquarium.

Gasping air (Surfacing behavior): fish rise to the water’s surface and gasp air, presumably as a result of the low aquarium’s dissolved oxygen content (Noga,^2010^).

Middle crossing test: This test measures how many fish cross the tank midline during a three minute period (Scott et al. 2003).

The operculum’s opening and shutting were counted over 60 s to determine the ventilation rate, which was then converted to opercular beats per minute (Leonard and Skov 2022).

### Blood and tissue sampling

On the 60th day of the experiment, fish were fasted for 24 h, and anesthetized using 100 mg/L benzocaine solution according to Neiffer (2009). Two blood samples were drawn from the caudal blood vessels of fifteen fish in each group. The first set was collected using EDTA-rinsed syringes for hematological assay. An automated hemocytometer (Hospitex Diagnostics, Sesto Fiorentino, Italy) was used for counting various blood cells. The second set was aspirated without using an anticoagulant for further serum isolation. These samples were then subjected to centrifugation for 15 min at 1500 rpm. The isolated serum samples were preserved at −20 °C to be employed for biochemical assessment. Nine brain specimens from each group were used for histopathological procedures. Nine additional brain samples from each group were excised and stored in Eppendorf tubes containing Trizol at − 80 °C for stress/apoptotic-related gene expression analysis.

### Stress mediators and lipid profile assay

The serum cortisol and glucose levels were assessed by the methods described by Saliu et al. (2017), Trinder (1969). The calorimetric technique previously released by Allain et al. (1974), and McGowan et al. (1983) was used for measuring the lipid profile markers including, cholesterol, and triglycerides, respectively.

### Gene expression of stress-related genes

A quantitative real-time polymerase chain reaction was employed to assess the impact of dietary SCN on the expression of stress and apoptotic-related genes in the brain of Nile tilapia (10 samples per group). qPCRs were performed in a Rotor-Gene Q 2plex Real-Time PCR System (Qiagen, Germany) using TOPreal™ qPCR 2X PreMIX (SYBR Green with low ROX) (Cat. # *p725* or *p750*) (Enzynomics, Korea) per the manufacturer’s guidelines, The total RNA from the tissue was isolated in 1 mL of Trizol reagent (Qiagen, Germany). cDNA synthesis and qPCR analysis followed protocols outlined by Khamis et al. (2021). Five stress/apoptotic-related genes (*hsp-70*,* tgf-β*,* tp53*,* chop*, and *bip/grp78*) were analyzed in qPCR using specific primers described by Livak and Schmittgen (2001) (Sangon Biotech, Beijing, China); Gapdh was employed as a reference housekeeping gene (supplementary file)(Table 2).

**Table 2:** Sequences of oligonucleotide primers and conditions for real-time PCR

Gene		Primer sequences	NCBI accession no.	PRODUCT SIZE (BP.)	Efficiency
*hsp70*	F R	GGGCGTCGACTTCTACACTT TCTGCCCCTTGTCCAGTTTG	GQ386813.1	125	98.52
*chop*	F R	GTGCCAAACTTGGAGCTGTG CTGAAGGTGCTCTTCCCCAG	XM_003438106.5	182	99.67
*bip/grp78*	F R	GTCGAGCAGATTGGAGAGCA CAGTCCCCACGTTCTCCTTC	XM_019361056.2	116	100.5
*tp53*	F R	AAAGGAGGAGGGGGAGAGAC CATGACCTGCTGTACTGGGT	XM_025905404.1	130	94.5
*tgf-b*	F R	GAACTTCGGCGGTACTGTGA CTGTCCGTTGTGTCAGTGGA	NM_001311325.1	200	93.61
*gapdh*	F R	CATGGGTGTCAACCACGAGA CAGTTGGTTGTGCAGGAAGC	XM_005455438.3	72	97.45

*hsp70*: Heat shock protein 70; *tgf-b*: transforming growth factor beta; *tp53*: tumor protein 53; *bip/grp78* (Glucose-regulated protein 78), and *chop*: the transcription factor C/EBP homologous protein; *gapdh*: Glyceraldehyde- 3-phosphate dehydrogenase,

### Histopathological examination

Histopathological investigations were conducted on the brain obtained from representative 5 tissue specimens that were randomly collected per group. The brain samples were promptly fixed in 10% buffered neutral formalin, dehydrated in gradually increasing ethanol, cleared in xylene, and embedded in paraffin. A microtome (Leica^®^, Wetzlar, Germany) was used to slice paraffin blocks at a thickness of 5 μm. Hematoxylin and eosin (H&E) stains were then applied to the sections. A Ceti England microscope with a digital camera (AmScope) was used to record images of sections during microscopic analysis (Suvarna et al. 2013). All measurements were obtained from the images using the AmScopeToupView software version 3.7 (AmScope, United States). A scoring scale was used to quantify tissue changes observed under a microscope, aiding in the assessment of disease severity and treatment effects.

### Statistical analysis

The data was analyzed using SPSS version 22.0 and a one-way analysis of variance (ANOVA). The Kolmogorov-Smirnov test was used to check for normality, and Levene’s test was used to determine if the variances were homogeneous before moving on to statistical evaluation, no departure from these assumptions was recorded for any of the traits observed in this study. Tukey’s post hoc test was used to conduct post hoc comparisons between groups. Significant at the level of *P <* 0.05, the findings were presented as the mean plus or minus the standard error of the mean (SEM).

## Results

### Characterization of SCN

**Fig. 1:**
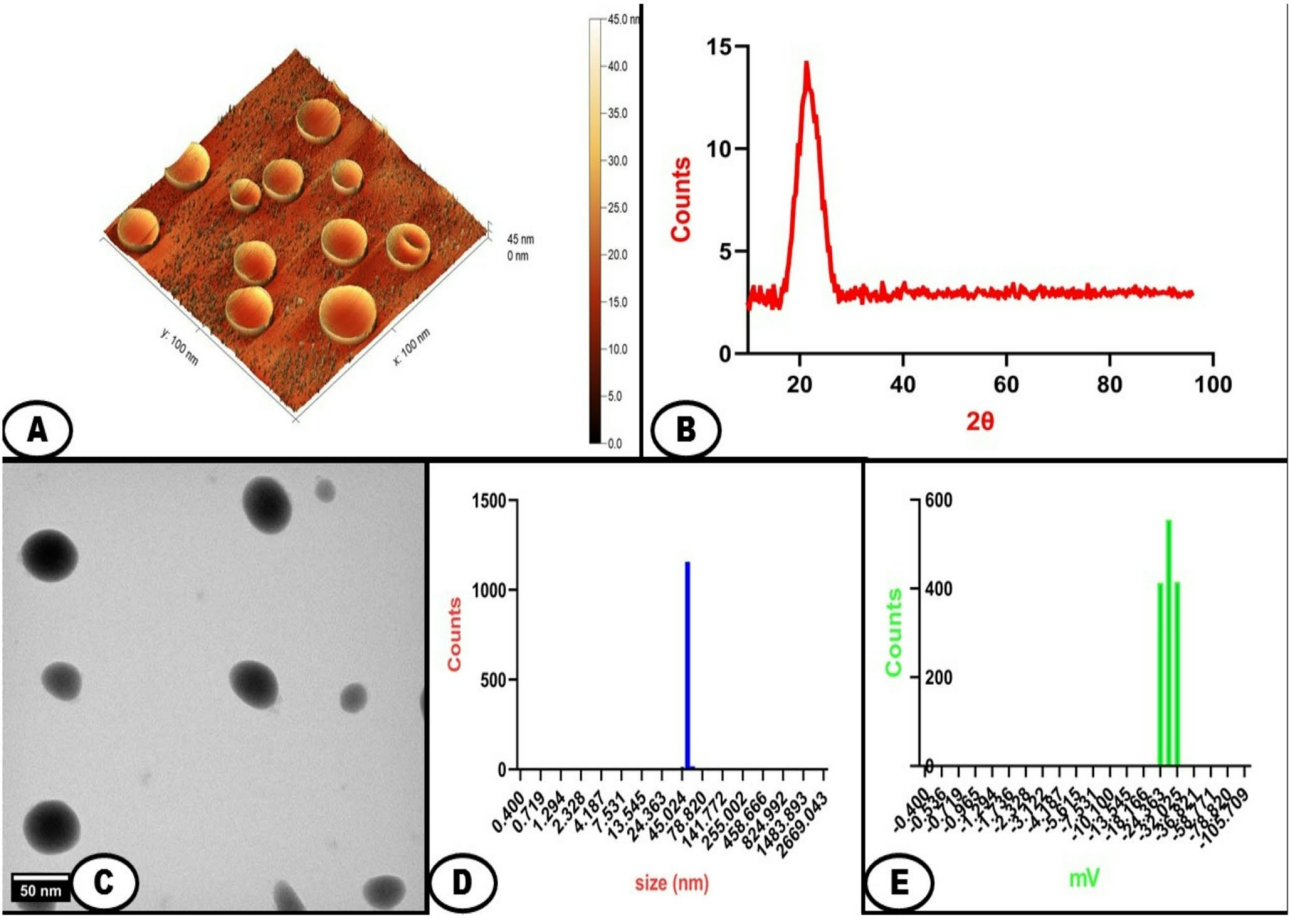
Morphological and physicochemical characterization of Spirulina-coenzyme Q10 nanoemulsion (SCN). (**A**) Atomic Force Microscopy topographical image showing spherical particles with smooth surface morphology; (**B**) X-Ray Diffraction patterns comparing pure Spirulina powder (upper trace) and SCN formulation (lower trace) showing transition from crystalline to amorphous state; (**C**) Transmission Electron Microscopy image revealing spherical particles with distinct boundaries; (**D**) Dynamic Light Scattering size distribution profile showing narrow size distribution; (E) Zeta potential distribution profile showing negative surface charge (*n* = 3, mean ± SD)

Spirulina with coenzyme Q10 nanoemulsion (SCN) exhibited spherical particles with smooth topography. The height measurements from Atomic Force Microscopy (AFM) indicated a size range of 43–47 nm, corroborating the slight size increase observed in DLS and electron microscopy analyses (Fig. 1A). The nanoemulsion maintained its particle size and PDI values when incorporated into the fish diet and stored at 4 °C for the duration of the experiment, with less than 5% change in mean particle diameter. The X-ray diffraction patterns of SCN provided crucial insights into their structural characteristics at the molecular level, revealing a primary broad halo centered at approximately 2θ = 22° (two-theta angle in X-ray diffraction notation). The absence of distinct Bragg peaks corroborates the amorphous state of this formulation (Fig. 1B). XRD analysis was performed on both pure Spirulina powder and the SCN formulation using a Rigaku SmartLab X-ray Diffractometer. Crystallinity information is crucial for predicting the dissolution behavior and bioavailability of active compounds in vivo. Pure Spirulina powder showed characteristic crystalline peaks at [insert specific peaks]. At the same time, the SCN formulation displayed a primary broad halo centered at approximately 2θ = 22° with the absence of distinct Bragg peaks, confirming the amorphous state of the nanoemulsion. This amorphous nature is advantageous as it typically results in enhanced dissolution rates and improved bioavailability compared to crystalline forms, which is particularly important for the therapeutic efficacy of the active compounds in aquaculture applications. Transmission Electron Microscopy images of the SCN revealed spherical particles with distinct boundaries (Fig. 1C). The average particle size from TEM analysis was 45.3 ± 3.2 nm (*n* = 100), with minimum and maximum diameters of 38.7 nm and 53.6 nm, respectively. Dynamic Light Scattering (DLS) Analysis of SCN exhibited an average hydrodynamic diameter of 45 nm, as evidenced by a single, sharp peak in the size distribution profile. This result indicates the successful formation of nano-sized droplets, well within the desirable range for nanoemulsions (typically 20–200 nm). The narrow width of the peak suggests a high degree of size uniformity among the droplets, which is crucial for ensuring consistent behavior and performance in biological systems (Fig. 1D). Spirulina-coenzyme Q10 nanoemulsion exhibited a zeta potential of −32 mV, as evidenced by a sharp, symmetrical peak in the distribution profile. This negative surface charge is indicative of the presence of anionic species at the oil-water interface of the nanoemulsion droplets (Fig. 1E). The high absolute zeta potential value (−32 mV) indicates excellent electrostatic stabilization. The zeta potential measurements were performed in triplicate using a Malvern Zetasizer Nano ZS. Results are presented as mean ± standard deviation (*n* = 3). The three bars in Fig. 1E represent three independent measurements to ensure reproducibility of the results.

### Behavioral responses

Results for all evaluated parameters in the group maintained at the control temperature (25 °C) and the tested high temperature (32 °C)are reported in Table 3. The ventilation rate (opercular movement), surfacing frequency, and surface swimming of Nile tilapia reared at 32 °C and fed the base diet (SCN0) were significantly higher than that of fish reared at 25 °C (C25) (*P <* 0.05). The feeding activity and middle swimming behavior were significantly lower (*P* < 0.05) in the heat-stressed group compared to the control (C25). Aggression indicators such as approach, chasing, and mouth pushing, did not differ significantly between groups (*P* > 0.05). Altogether, adding SCN to Nile tilapia diets significantly (*P <* 0.05) enhanced most behavioral parameters, particularly in the SCN20 and SCN40 groups (Table 3).

**Table 3:** Effect of heat stress (32 °C water temperature) and spirulina Co-enzyme Q10 nano-emulsions (SCN) fortified diet on the behavioral response of nile tilapia for 60 days

Behavioral patterns	Experimental groups						*P* value
Items		C25	SCN0	SCN10	SCN20	SCN40	
Feeding frequency		13.98 ± 0.19^a^	12.7 ± 0.42^b^	13.19 ± 0.41^ab^	13.79 ± 0.28^a^	14.06 ± 0.13^a^	0.018
Surfacing frequency		1.9 ± 0.31^b^	4.7 ± 0.36^a^	3.8 ± 0.29^a^	2.5 ± 0.30^b^	2.6 ± 0.30^b^	0.000
Swimming	Surface	3.2 ± 0.13^b^	5.8 ± 0.41^a^	5.7 ± 0.21^a^	3.8 ± 0.32^b^	3.9 ± 0.23^b^	0.000
	Middle	12.6 ± 0.58^a^	6.9 ± 0.64^c^	7.5 ± 0.71^c^	9.4 ± 0.61^b^	10 ± 0.57^b^	0.006
	Bottom	0.8 ± 0.20	0.1 ± 0.1	0.4 ± 0.51	0.4 ± 0.51	0.6 ± 0.51	0.550
Aggressive	Approach	2.6 ± 0.22	1.1 ± 0.27	1.2 ± 0.41	1.6 ± 0.71	2.2 ± 0.57	0.140
	chasing	14.01 ± 0.8	12.7 ± 0.63	11.5 ± 0.81	12.4 ± 1.08	13.7 ± 0.78	0.233
	Mouth pushing	3.2 ± 0.24	2 ± 0.61	2.4 ± 0.56	2.4 ± 0.42	2.9 ± 0.27	0.371
Resting		2.3 ± 0.49	0.9 ± 0.31	1.3 ± 0.44	1.4 ± 0.33	2.00 ± 0.39	0.619
No. of Middle crossing		18.4 ± 1.03	14.1 ± 1.95	14.5 ± 1.43	18.1 ± 1.04	18.2 ± 0.92	0.117
Opercular movement		78 ± 1.07^c^	96.2 ± 1.17^a^	83.1 ± 0.93^b^	83.1 ± 1.01^b^	81.1 ± 0.84^b^	0.000

C25 = Control fish were fed on a basal diet at 25 °C. SCN0 = Fish were fed on a basal diet at heat stress (32 °C). SCN10 = Fish were fed a diet supplemented with 10 mg Spirulina coenzyme nanoemulsion at heat stress (32 °C). SCN20 = Fish were fed a diet supplemented with 20 mg SCN at heat stress (32 °C). SCN40 = Fish were fed a diet supplemented with 40 mg SCN at heat stress (32 °C). Means in the same column with different superscripts are significantly different (*P* ˂ 0.05)

### Modulations in blood profile

Table 4 presents the effects of SCN supplements on hematological markers of heat-stress *O. niloticus*. Fish reared at 32 °C and fed the base diet (SCN0) had considerably lower mean values for RBCs, Hb, HCT, MCV, MCH, and MCHC compared to the other groups (*p <* 0.05). In contrast, blood indices were markedly enhanced *(p <* 0.05), in heat-stressed groups, in response to dietary incorporation with 20 and 40 mg/kg SCN, compared to the stressed, non-supplemented group (SCN0). The leukogram profile indexes (WBCs, monocyte%, lymphocyte%, heterophil%) were notably decreased *(p <* 0.05) in heat-stressed groups (SCN0), with significant improvement in SCN40 and SCN20 groups which did not differ significantly from the C25 group *(p >* 0.05).

**Table 4:** Effect of heat stress (32 °C water temperature) and spirulina Co-enzyme Q10 nano-emulsions (SCN) fortified diet on hematological parameters of nile tilapia for 60 days

Parameters			Experimental groups			*P* value
	C25	SCN0	SCN10	SCN20	SCN40	
RBCs (10^6^/µl)	2.11 ± 0.01^a^	1.28 ± 0.01^c^	1.55 ± 0.08^b^	2.07 ± 0.00^a^	2.1 ± 0.03^a^	0.000
HCT (%)	28.54 ± 0.00^a^	18.44 ± 0.00^c^	26.57 ± 0.9^b^	27.5 ± 0.28^ab^	28.00 ± 0.00^a^	0.000
Hb (g/dL)	11.05 ± 0.31^a^	6.45 ± 0.08^c^	8.9 ± 0.98^b^	10.25 ± 0.14^ab^	10.2 ± 0.7^ab^	0.000
MCV (fl.)	145 ± 2.59^a^	134 ± 1.15^b^	135 ± 0.57^b^	142.5 ± 3.17^a^	142.5 ± 0.29^a^	0.006
MCH (pg)	52.3 ± 0.23^a^	48.6 ± 0.11^b^	49.5 ± 0.63^ab^	51.95 ± 0.31^a^	52.45 ± 1.81^a^	0.032
MCHC (g/dl)	38.8 ± 0.17^a^	34.1 ± 0.02^b^	34.75 ± 1.24^b^	35.9 ± 0.43^b^	38.7 ± 1.15^a^	0.004
WBCs (10³/µl)	80.22 ± 0.53^a^	86.54 ± 089^c^	83.93 ± 0.61^bc^	82.21 ± 0.19^bc^	81.96 ± 1.1^b^	0.001
Monocyte %	22.00 ± 3.23^a^	8.9 ± 0.51^c^	10.1 ± 1.06^bc^	15.95 ± 0.54^ab^	21.6 ± 3.17^a^	0.003
Lymphocytes %	85.33 ± 0.33^a^	68.00 ± 4.04^c^	77.00 ± 2.3^b^	82 ± 1.15^ab^	80.5 ± 1.44^ab^	0.000
Heterophil %	10 ± 1.15^a^	6.5 ± 0.28^b^	7.5 ± 0.28^ab^	9.5 ± 1.4^ab^	9.5 ± 0.28^ab^	0.096

Values are mean ± SE for three samples/replicate (*n* = 10/group), values are not sharing a common superscript letter differ significantly at *p* < 0.05

RBC: erythrocyte count; Hb: hemoglobin concentration; HCT: hematocrit value; MCV: mean corpuscular volume, MCHC: mean corpuscular hemoglobin concentration; WBC: leukocyte count

### Stress mediators assay

The serum stress indicators (cholesterol, triglycerides, glucose, cortisol) were significantly higher (*p <* 0.05) in the SCN0 groupthan in SCN40, SCN20, SCN10 and C25 groups. The C25 group showed the lowest values of serum stress indicators; the level of triglycerides and glucose did not differ significantly between this group and the SCN40 group (Table 5).

**Table 5:** Effect of heat stress (32 °C water temperature) and spirulina Co-enzyme Q10 nano-emulsions (SCN) fortified diet on serum lipid profile stress indicators and the histology lesions score of the brain tissue of nile tilapia for 60 days

	Parameters	Experimental groups					P value
		C25	SCN0	SCN10	SCN20	SCN40	
Serum	Cholesterol (mg/dl)	65.52 ± 1.83^e^	127.88 ± 0.85^a^	100.04 ± 0.62^b^	81.99 ± 0.79^c^	76.22 ± 0.94^d^	0.000
	Triglycerides (mg/dl)	52.43 ± 1.53^c^	71.21 ± 1.29^a^	58.95 ± 1.92^b^	57.55 ± 3.9^bc^	57.42 ± 0.83^bc^	0.000
	Glucose (mg/dl)	48.45 ± 0.059^c^	93.92 ± 0.94^a^	69.62 ± 0.54^b^	50.95 ± 1.38^c^	50.00 ± 0.00^c^	0.000
	Cortisol (ng/mL)	11.18 ± 0.62^e^	22.14 ± 0.08^a^	19.1 ± 0.7^b^	16.3 ± 0.38^c^	13.92 ± 0.32^d^	0.000
Brain histology	Vascular congestion	0.00 ± 0.00^b^	8.00 ± 1.33^a^	4.00 ± 1.63^b^	2.00 ± 1.33^b^	3.4 ± 1.52^b^	0.002
	Perivascular edema	0.00 ± 0.00^b^	5.00 ± 1.66^a^	4.00 ± 1.63^ab^	2.00 ± 1.33^ab^	3.00 ± 1.52^ab^	0.121
	Neuronal pyknosis	0.00 ± 0.00^c^	28.00 ± 5.92^a^	12.00 ± 1.33^b^	3.00 ± 1.63^c^	4.00 ± 1.63^bc^	0.000
	Perineuronal vacuolation	2.00 ± 4.33^d^	45.00 ± 5^a^	22.00 ± 4.6^b^	11.00 ± 1.00^cd^	15.00 ± 1.66^bc^	0.000

Means in the same column with different superscripts are significantly different (*P* ˂ 0.05)

### Expression of stress/apoptotic-related genes

**Fig. 2:**
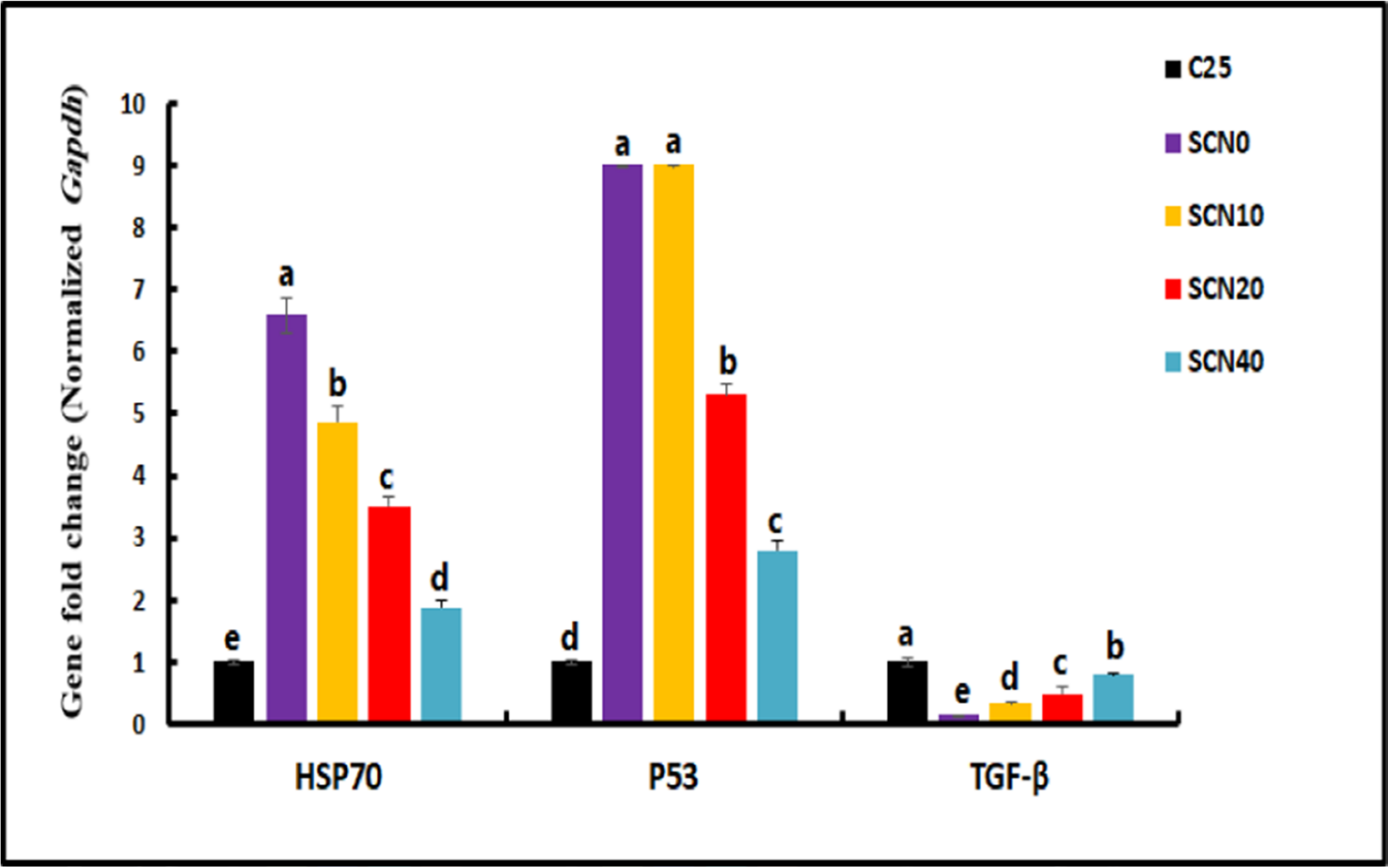
Changes in heat-stressed *Oreochromis niloticus* brain mRNA expression of heat shock protein 70 (*hsp70*), tumor protein p53 (*p53*), and transforming growth factor beta (*tgf-b*) genes after 60 days of a diet supplemented with SCN. Data represented as mean ± SE, *N* = 10 for each group. Bars with distinct letters (**a**, **b**, and **c**) indicate significant differences at *p <* 0.05

**Fig. 3:**
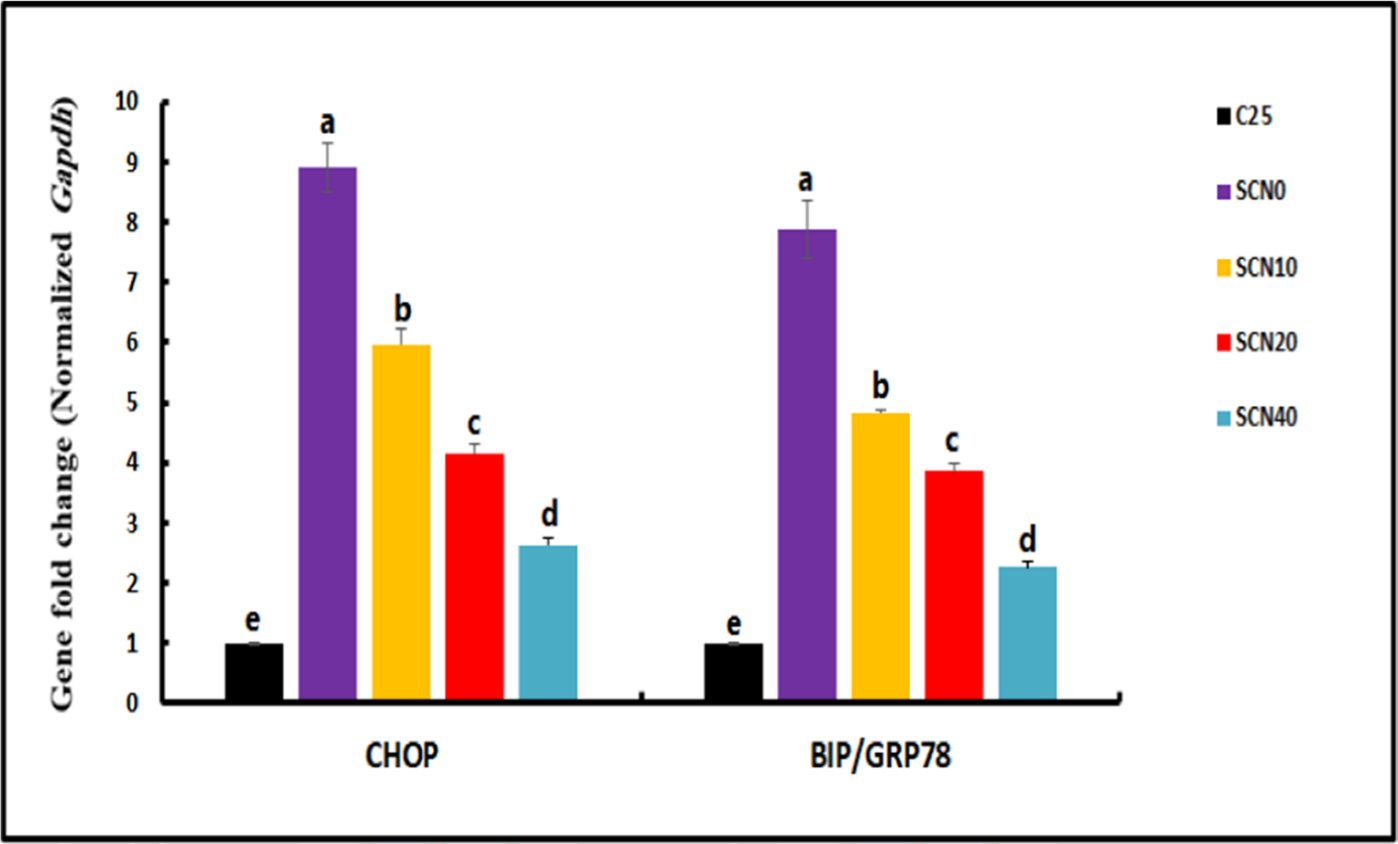
Changes in heat-stressed *Oreochromis niloticus* brain mRNA expression of C/EBP homologous protein (*chop*) and Glucose-regulated protein 78 or Binding immunoglobulin protein (*bip*/*grp78*) genes after 60 days of a diet supplemented with SCN. Data represented as mean ± SE, *N* = 10 for each group. Bars with distinct letters (**a**, **b**, and **c**) indicate significant differences at *p <* 0.05

The expression profile of stress**/**apoptotic-related genes *hsp-70*,* tgf-β*,* tp53*,* chop* and *bip* in the brain of Nile tilapia of all groups are presented in Figs. 2 and 3. There was a significant downregulation (*p <* 0.05) in the expressions of *hsp-70*,* tp53*,* chop*, and *bip* genes in SCN- incorporated groups compared to the SCN0 group, and the downregulation was more pronounced as the level of supplementation increased; Accordingly, the SCN40 group represented the lowest value, closest to the control. In contrast, SCN-Supplemented groups, displayed significantly higher levels (*p <* 0.05) of *tgf-b* than SCN0 group, placing them closer to the control groups as the level of supplementation increased (Fig. 2).

### Histopathological findings of brain

**Fig. 4:**
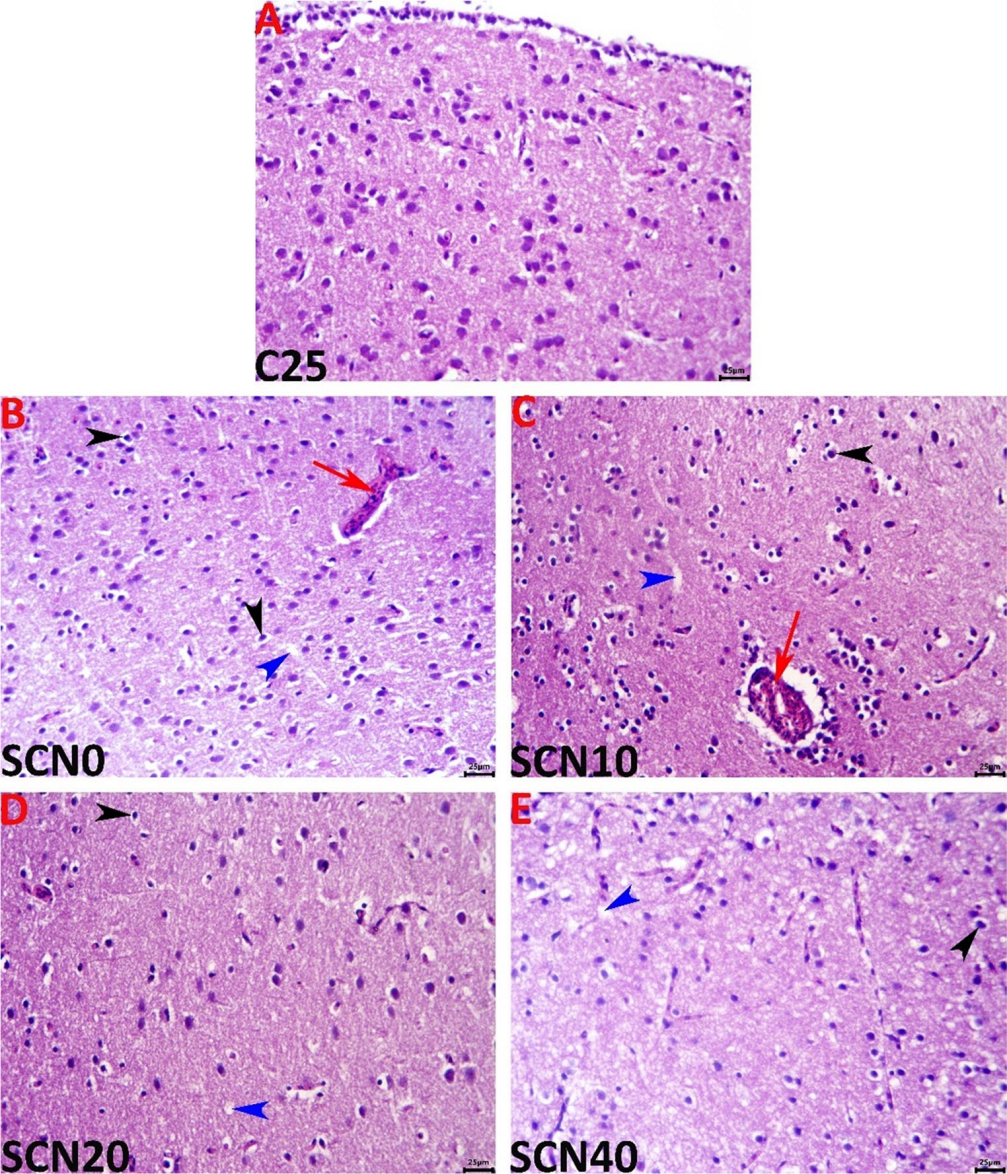
Representative light micrographs of the H&E-stained cerebral tissue sections show normal histological pictures in the C25 group, and varying degrees of vascular congestion with perivascular edema (red arrows), neuronal pyknosis associated with perineuronal vacuolation (black arrowheads), and neuropil microcavitations (blue arrowheads) in the SCN0, SCN10, SCN20, and SCN40 groups. The scale bar equals 25 μm

Histological features of the brain of Nile tilapia sampled from the various experimental groups are presented in Fig. 4. The examined cerebral tissue sections of the C25 group showed no sign of histopathology. Rearing tilapias in aquariums at 32 °C for 60 days incited minor but significant encephalopathic alterations in the SCN0 group such as vascular congestion, perivascular edema, neuronal pyknosis associated with perineuronal vacuolation, and neuropil microcavitation (Fig. 4). Supplementing the diet of heat-stressed tilapia with a nano-emulsion of spirulina and Coenzyme Q10 demonstrated dose-dependent neuroprotective effects with a reduction of the symptoms described above. The most favorable results were achieved with the 20 mg supplemented diet, then the 40 mg diet, and lastly, the 10 mg diet. Despite these observed neuroprotective effects of the SCN, the brain tissues did not maintain their normal histology in any supplemented group, and few encephalopathic lesions were still evident in all groups represented mainly by vascular congestions, and neuronal pyknosis including perineuronal vacuolation in the SCN10 group, neuronal pyknosis in the SCN20 group, and neuronal pyknosis and neuropil microcavitations in the SCN40 group. The encephalopathic alterations of all groups were summarized in Table 5.

## Discussion

Climate change contributes to extreme weather events, which have profound implications for aquatic ecosystems and may have multiple effects on the aquaculture sector. Behavior, survival, nutrition, development, and every other facet of a fish’s life can be impacted by alterations in a river’s temperature regime (Mahmoud et al. 2020). To lessen the impact of heat stress on aquaculture systems, nutritional modification and supplementation of aquafeeds are important (Abu-Zahra et al. 2025). Currently, the utilization of nanoemulsions in aquafeeds has garnered significant global interest, exhibiting beneficial benefits on stress alleviation, enhanced disease resistance, and overall health improvement (Ahmed et al. 2025). This study is the first to document that the incorporation of SCN into the diet mitigated the adverse effects induced by heat stress in Nile tilapia.

The morphology, content, distribution of particle dimensions, diameter of pores, surface area, and surface charge of SCN were all assessed using several procedures as AFM, TEM, XRD, DLS, and zeta potential measurements. SCN exhibited spherical particles with smooth topography. The little increase in size noted in DLS and electron microscopy analyses was supported by the height measurements from AFM which showed a size range of 43–47 nm. The nanoemulsion maintained its particle size when incorporated into the fish diet and stored at 4 °C for the duration of the experiment with less than 5% change in mean particle diameter. The nanoemulsion formation process relies on the reduction of interfacial tension between oil and water phases through the use of surfactants, combined with high-energy methods to achieve nano-sized droplets. The selection of extra virgin olive oil as the oil phase was based on its biocompatibility and ability to solubilize Coenzyme Q10, while Tween 80 was chosen as a non-ionic surfactant due to its low toxicity and excellent emulsifying properties. The high-speed homogenization creates initial droplet breakdown, while ultrasonication provides the additional energy required to achieve nanoscale dimensions through cavitational forces that further reduce particle size and improve size distribution uniformity.

Fish behavior is a reflection of how fish respond to their surroundings (Cooke 2016; Elsayyad et al. 2024). According to Martins et al. (2012), behavioral alterations related to aggression, and swimming have been linked to stresses and poor welfare in aquaculture conditions. In our experiment, feeding activity and the middle crossing indicator were sharply decreased in the heat-stressed fish group. The reduced feeding rate of Nile Tilapia under excessive water temperature was already reported in a previous study by Dawood et al. (2019), while alterations in swimming behavior and impaired locomotion were reported following temperature shifts by Donaldson et al. (2008). This study also reported an increase in surfacing frequency and surface swimming in heat-stressed fish. The latter findings could be a result of the aquarium’s low dissolved oxygen level, as dissolved oxygen in an aquatic system decreases as the water temperature rises because water has a lower capacity to hold oxygen at higher temperatures, as also noted by Conte (2004) and observed by Islam et al. (2020). Our results revealed that aggressive behavior was not affected by heat stress. Gherardi et al. (2013) also noted that fish aggressiveness was unaffected by changes in water temperature. In this study, fish that were fed a diet that included SCN had an increase in feeding frequency and an improvement in swimming behavior as compared to the group fed a basal diet and reared at 32 °C; the greatest improvements were observed when the supplement was fed at 20–40 mg/kg. An earlier investigation confirmed that dietary supplementation with Coenzyme Q10 led to substantial enhancement in all neurobehavioral indicators of stressed Nile tilapia (Khalil et al. 2022). Asgari et al. (2025) demonstrated that Rainbow Trout feed efficiency was enhanced when a diet containing more than 20 mg CoQ10/kg. The possible reasons behind the increased feeding frequency and activity caused by CoQ10 include vitamin E re-synthesis (Linnane et al. 2007), changes in microflora diversity, anti-inflammatory effects (Schmeltzer et al., 2008), and improved digestive enzyme activity (El Basuini et al. 2021). In addition, Henricksen (1999) found that fish performance might be effectively improved by CoQ10’s effect on insulin, glucagon, and cortisone. Overall, these studies suggest that CoQ10 is an effective defense mechanism against neurotoxic effects and neurobehavioral abnormalities in Nile tilapia. Two possible mechanisms that underlie the neuroprotective effects of CoQ10 are its antioxidant activities and its ability to enhance AChE (Khalil et al. 2022). Nanoparticulation seems to enhance the original physicochemical features of CoQ10 and spirulina (Eftekhari et al. 2018). Elabd et al. (2020) showed that supplementing the diet of Nile Tilapia with spirulina nanoparticles increased their antioxidant activity and resilience to temperature changes, and Eissa et al. (2024) reported that supplementing Nile tilapia with SP increased intestinal enzyme activity, which in turn increases feed consumption and decreases nutrient retention. The high concentration of bioactive compounds in spirulina is believed to be responsible for the potent cytoprotective effect. These bioactive compounds include polysaccharides, γ-linolenic acid, polyunsaturated fatty acids, and pigments such as total carotenoids, chlorophyll, β-carotene, zeaxanthin, and phycocyanin (Awad et al. 2022). As a microalgae involved in phytoremediation, SP has been found to mitigate the harmful effects of polyethylene nanoparticles on crayfish (Mokhtar et al. 2026). The prolonged presence of spirulina nanoparticles in the bloodstream strengthens the availability of these compounds, making them more effective than their conventional form (Hill and Li 2017).

Hematological indices convey important information on the physiological responses, metabolic processes, and stressors experienced by fish (Shaalan et al. 2025). Herein, heat-stressed fish displayed significant anemia and leukopenia. A possible explanation for the dramatic drop in hemoglobin and red blood cell concentrations in this study is a failure of the hematological system induced by heat stress. Changes in the Hb and RBC concentration in response to thermal stress were also reported in cichlids (Carvalho and Fernandes 2006) and striped catfish (Islam et al. 2019). Ashaf-Ud-Doulah et al. (2019) noticed significant alterations to hemato-biochemical indices and morphology of erythrocytes and leucocytes in heat stressed Indian major carp, rohu *Labeo rohita*. Also, by influencing changes in the lipid bilayer and energy storage, heat stress can affect cellular shape and blood cell count (Islam et al. 2022). In this study, the blood indices of heat-stressed Nile tilapia that were fed a diet supplemented with SCN showed a significant improvement of blood indices as compared to groups fed only the basal diet. The improved hematological indicators in SCN-supplemented groups suggest that hemosynthesis and erythropoiesis increased in response to the treatment and benefited the health of heat-stressed Nile tilapia. These results confirm observations by Eissa et al. (2024) who reported that the red blood cell and white blood cell counts, hemoglobin and PCV concentrations, monocytes, and neutrophils of Nile tilapia-fed SP diets were all increased in groups fed *Spirulina platensis* in comparison to controls. Also, protecting from anemia under heat stress circumstances is possible with the help of SP-enriched NPs (El-Ratel et al. 2023). The positive effects of SP diets may be due to phycocyanin, as this pigment was shown to stimulate the stem cells of the bone marrow, which promotes the immune response and red blood cell production in fish, resulting in increases of Hb, MCH, MCHC, and HCT in Oscar fish (Mohammadiazarm et al. 2021). Boosting total red blood cells with the help of CoQ10 allows for an increase in hemoglobin levels in rabbit (Rakha et al. 2026). Furthermore, Littarru et al. (1994) mentioned that hemolysis caused by a free radical initiator was found to be less likely to occur in erythrocytes that had previously been enriched with exogenous CoQ10.

Prolonged exposure to high temperatures is often associated with higher blood cortisol levels (Kim et al. 2019). When fish are subjected to extreme temperatures, their energy demands rise. In response, they break down their glycogen stores, which raises their serum glucose concentration. This gives them more energy to deal with the heat and keep their body in balance (Long et al. 2023). In this study, dietary SCN supplementation of heat stressed Nile tilapia almost restored the serum levels of stress indicators to their normal values. Hence, the lowest levels of these stress markers were recorded in the SCN20, and SCN40 groups compared to SCN0. Similarly, Awad et al. (2022) recorded a significant decrease in cholesterol and triglyceride levels in the spirulina-supplemented group. Spirulina may reduce cholesterol levels by increasing the activity of the lipase enzyme, which aids in lipoprotein breakdown (Mahmoud et al. 2018). It’s interesting to note that fish-fed diets supplemented with CoQ10 had lower glucose levels than fish-fed diets without the supplement in this study, which may be related to the ability of CoQ10 to enhance insulin sensitivity because it regulates the insulin and adiponectin receptors, tyrosine kinase (TK), phosphatidylinositol kinase (PI3K), and glucose transporters. Furthermore, the noticeable drop in the levels of triglyceride and cholesterol, is most likely due to Coenzyme Q10’s efficiency in inducing hypocholesterolemia in treated fish (Raeisi-Zeydabad et al. 2017).

Temperature stress not only impairs metabolism but also activates regulatory networks tasked with reducing and avoiding further harm. Due to their capacity to protect cells and tissues from structural damage during successive exposures to stress, *hsps* play crucial roles in long-term stress adaptation (Reyes-Lopez et al., 2018). Herein, our findings suggest that heat stress upregulates *hsp70*, suggesting that Nile Tilapia relies on *hsp* genes to react to heat stress. *hsp* protein upregulation was also observed in the livers and kidneys of rainbow trout exposed to heat stress (Huang et al. 2018; Li et al. 2017). It has been found that heat shock proteins (*hsps*) can repair and prevent cellular damage caused by protein denaturation at high temperatures in their role as molecular chaperones (Werner et al. 2006). Also, the liver of *Triplophysa siluroides* showed an upregulation of several members of the *hsp* family after thermal stress (Chen et al. 2023).

Tumor protein *p53* is triggered by many physiological cues, including oxidative stress, damage to the DNA, and glucose restriction that can result from exposure to several developmental adversities, including hyperthermia (Hosako et al. 2007). In this study, heat-treated fish showed an upregulation of *tp53*. Enhanced expression of *bip*, and *chop* was also observed. Following heat stress, the endoplasmic reticulum (ER) undergoes unfolded protein response (UPR), which in turn causes an increase in the levels of the pro-apoptotic protein *chop* and the ER chaperone *bip* (*grp78*) (Almanza et al. 2019). As a protective mechanism against heat shock-induced endoplasmic reticulum stress, this UPR tries to normalize protein folding. On the other hand, acute or chronic stress can trigger apoptosis by activating *chop* through the UPR (Liu et al. 2017). Similary, the apoptotic rate and genes associated to apoptosis were both elevated in largemouth bass livers treated with heat stress (Zhao et al. 2022). *tgf-b* can enhance the natural process of eliminating damaged or abnormal cells from various healthy organs and tissues (Fabregat et al. 2016). Of note, the pathways of heat stress response in the brain mostly dealt with the production and secretion of cortisol, connections between neuroactive ligands and receptors, the *tgfb* signaling system, and the genes involved in *hsps* in *O. niloticus* (Wang et al. 2023). Furthermore, heat shock proteins, oxidative stress, neuronal activation, endoplasmic reticulum stress, and apoptosis are some of how the brain of *O. bidens* fish reacts to high temperatures (Li et al. 2024). This study indicated that administering 20–40 mg/kg of SCN can preserve brain integrity in Nile tilapia during heat stress by suppressing the expression of stress-related genes (*hsp70*, *tp53*,* bip*, and *chop*) and activating the *tgf-b* gene in brain tissue which indicates better anti-inflammatory and protective reactions in treated fish. One possible explanation for spirulina’s anti-genotoxic effects is the presence of the antioxidants phycocyanin and phycocyanobilin, which have potent anticyclooxygenase-2 and peroxidinitrite scavenging and DNA oxidative damage reduction capabilities (Bhat and Madyastha 2001). With continued encephalopathological regeneration, an additional mechanism by which spirulina extract inhibits the inflammatory cascade is by regulating inflammatory cytokines (Hussein et al. 2018) that help keep the brain and body healthy by preventing neurodegeneration and oxidative stress (Attiya et al. 2019). As an antioxidant, CoQ10 protects DNA from heat-induced oxidative damage and cellular phospholipids and mitochondrial membrane proteins from peroxidation, as well as neuroprotective function reported in other studies (Niklowitz et al. 2007).

According to Nakano et al. (2014), cold-blooded fish species suffered significant harm from high temperatures, creating metabolic stress in the body.

Throughout the study, the histopathological outcomes of heat stress were prevented in SCN groups compared to the SCN0 control after the 60-day challenge. Fish exposed to heat stress developed encephalopathic alterations such as vascular congestion, perivascular edema, neuronal pyknosis associated with perineuronal vacuolation, and neuropil microcavitation. Elevated water temperatures trigger an overabundance of reactive oxygen species (ROS), which can harm the phospholipid membranes of many biological components, leading to metabolic and inflammatory dysfunctions in the tissues (Alfons et al. 2023). On the other hand, Khalil et al. (2022) noted a normal histological structure of the brain of the CoQ10-supplemented *O niloticus* after 60 days of the experiment.

## Conclusions

In conclusion, under conditions of elevated water temperature (32 °C), Nile tilapia hematology and physiology were promoted by the addition of 20–40 mg/kg of SCN to the diet in the form of nanoemulsion. Moreover, it diminished cell apoptosis, suppressed inflammatory responses, and eased endoplasmic reticulum stress-related genes, thereby enhancing the recovery from heat-induced stress in Nile tilapia. And it modulates neuro-behavioral metrics and brain morphology, collectively aiding in the attenuation of heat-induced stress in Nile tilapia.

## Supplementary Information

Below is the link to the electronic supplementary material.

Supplementary Material 1 (DOCX 87.0 KB)

## Data Availability

The datasets generated or analyzed during the current study are not publicly available but are available from the corresponding author upon reasonable request.
