# Supplementary material for: Behavioral, physiological, encephalopathological alterations and endoplasmic reticulum stress/apoptotic-related gene transcription in reaction to heat stress in Oreochromis niloticus fish: impact of dietary supplements with Spirulina-Coenzyme Q10 nanoemulsion
Source: Vet Res Commun. 2026 Jan 21;50(2):122. doi: 10.1007/s11259-025-11017-y (PMC12823635; doi:10.1007/s11259-025-11017-y)

**Behavioral, physiological, encephalopathological alterations and endoplasmic reticulum stress/apoptotic-related gene transcription in reaction to heat stress in *Oreochromis niloticus* fish: Impact of dietary supplements with Spirulina-coenzyme Q10 nanoemulsion**

**Walaa El-Houseiny ^1,*^, Mohamed Elhady^1^, Shaimaa A. A. Ahmed^1^, Tarek Khamis^2,3^, Sameh H. Ismail^4^, Mohamed M.M. Metwally^5,6^, Wessam El-Shahat^1^**

^1^ Department of Aquatic Animal Medicine, Faculty of Veterinary Medicine, Zagazig University, Zagazig, 44511, Egypt

^2^ Department of Pharmacology, Faculty of Veterinary Medicine, Zagazig University, Zagazig, 44511, Egypt

^3^ Laboratory of Biotechnology, Faculty of Veterinary Medicine, Zagazig University, Zagazig 44511, Egypt

^4^ Faculty of Nanotechnology for Postgraduate Studies, Sheikh Zayed Branch Campus, Sheikh Zayed City, Cairo University,12588, Giza, Egypt

^5^ Department of Pathology and Clinical Pathology, Faculty of Veterinary Medicine, King Salman International University, Ras Sudr, Egypt

^6^ Department of Pathology, Faculty of Veterinary Medicine, Zagazig University, Sharkia, Zagazig, 44511, Egypt


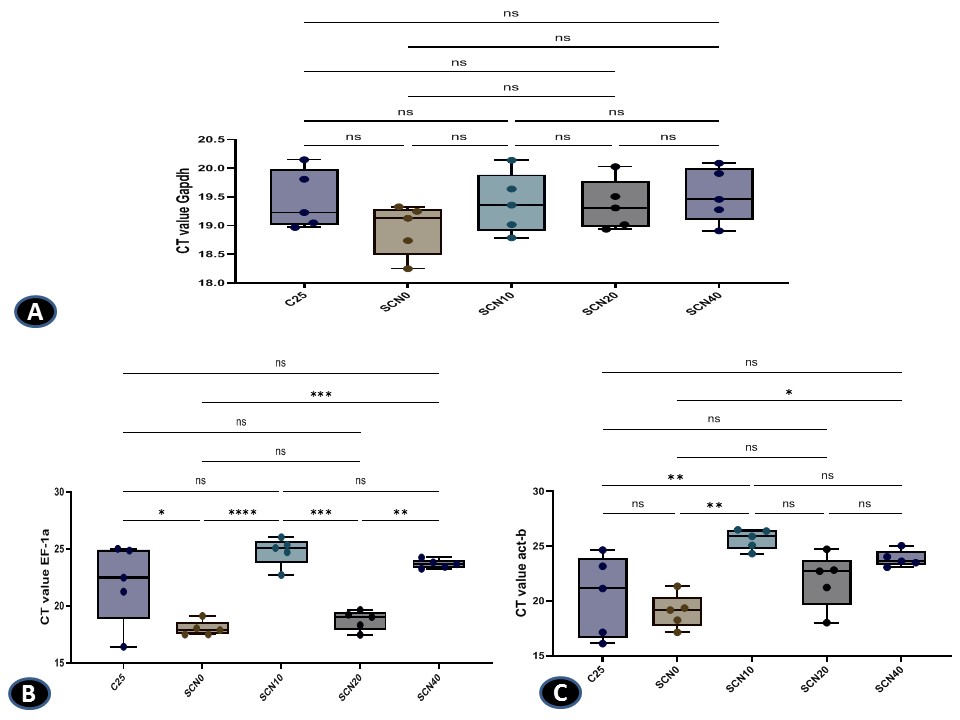

Supplement: Supplementary file 1 — Supplementary Material 1 (DOCX 87.0 KB) [file 11259_2025_11017_MOESM1_ESM.docx]
